# Supplementary material for: Ultrasensitive Nanophotonic Random Spectrometer with Microfluidic Channels as a Sensor for Biological Applications
Source: Nanomaterials (Basel). 2022 Dec 24;13(1):81. doi: 10.3390/nano13010081 (PMC9824005; doi:10.3390/nano13010081)
Supplement: Supplementary file 1 [file nanomaterials-13-00081-s001.zip › nanomaterials-2102233-supplementary.pdf]

Supplementary Materials: Ultrasensitive Nanophotonic Random Spectrometer with Microfluidic Channels as a Sensor for Biological Applications

Aleksei Kuzin [1,2,*,†], , Ilia Fradkin [3,4,†], Vasiliy Chernyshev [1], Vadim Kovalyuk [5,6], Pavel An [2,7], Alexander Golikov [2,5], Irina Florya [5], Nikolay Gippius [3], Dmitry Gorin [1] and Gregory Goltsman [6,7]

1. Optimization of Random spectrometer design

The sample consisted of 6 spectrometers six spectrometers of different hole diameter d (150 nm and 200 nm) and different radii R and hole quantity N (100 μm (5000), 150 μm (11450), 200 μm (20000)) have been varied. Every device estimated for the working performance by calculating the correlation matrix. Each element of the matrix was easily calculated by the relation $r_{ij} = \frac{\int I_i(\lambda) I_j(\lambda) d\lambda}{\sqrt{\int I_i^2 d\lambda \int I_j^2 d\lambda}}$. Based on this calculation the device configuration with the less ballistic mode channels was determined (see Figure S1). For next stage the spectrometer with optimal values for d = 150 nm, R = 150 μm and n = 11450 was chosen. However, they still suffer from not correct calibration. For the next step, exclusion of poorly calibrated spectrum region from the correlation calculation made for the improving correlation matrix values.

2. Dependence of sensitivity on temperature variation

After fabricating the random structure on the chip the speckle pattern is very stable due to no movement of scatterers (air holes etched into the optical layer). However the temperature variation can change the refractive index of Silicon Nitride material (Si_3N_4) for input optical radiation which will be the reason for the changing speckle pattern and as a consequence the sensitivity. In paper [1] authors demonstrated a simulation where the speckle pattern was generated for a fixed input wavelength (λ = 1512.5 nm) at different temperatures by changing the refractive index of silicon (Si). The thermo-optic coefficient (dn/dT) of Si, is approximately 1.8×10^{-4} K^{-1} [2]. As a result, considering up to $\pm$ 4 K change of temperature, the input wavelength can be recovered to the accuracy of the spectral resolution ($\sim$ 0.5 nm). In the case where Si_3N_4 material is used as the waveguide layer the speckle pattern dependence on temperature will be less due to lower dn/dT for this material (2.51×10^{-5} K^{-1}) [3]. In any case, to compensate thermal variation the temperature stabilization techniques can be used for microcavity-based devices or device calibration at multiple temperatures.

References

1. Redding, B., Liew, S. F., Sarma, R., and Cao, H. Compact spectrometer based on a disordered photonic chip. *Nature Photonics* **2013**, *7(9)*, 746-751.
2. Komma, J., Schwarz, C., Hofmann, G., Heinert, D., Nawrodt, R., Thermo-optic coefficient of silicon at 1550 nm and cryogenic temepratures. *Applied Physics Letters* **2012**, *101*, 041905.
3. Elshaari, A. W., Zadeh, I. E., Jöns, K. D., and Zwiller, V. Thermo-optic characterization of silicon nitride resonators for cryogenic photonic circuits. *IEEE Photonics Journal* **2016**, *8(3)*, 1-9.

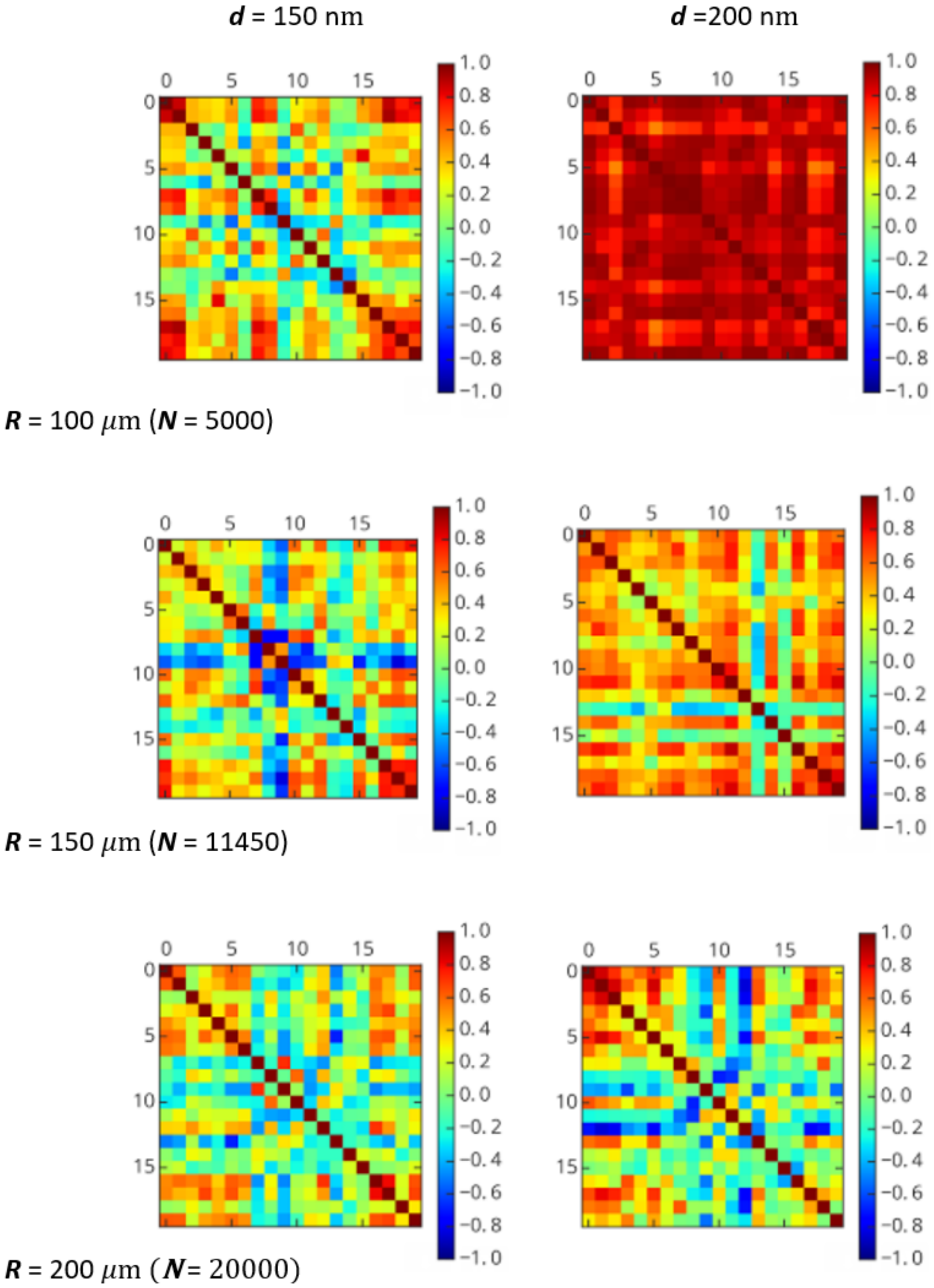

Figure S1. Correlation matrices for spectrometers of different size with holes of various radii; *R* is a Random spectrometer radius, *d* is a hole diameter, *N* is a hole quantity.
